# X-FIDO: An Effective Application for Detecting Olive Quick Decline Syndrome with Deep Learning and Data Fusion

**DOI:** 10.3389/fpls.2017.01741

**Published:** 2017-10-10

**Authors:** Albert C. Cruz, Andrea Luvisi, Luigi De Bellis, Yiannis Ampatzidis

**Affiliations:** ^1^Department of Computer and Electrical Engineering and Computer Science, California State University, Bakersfield, Bakersfield, CA, United States; ^2^Department of Biological and Environmental Sciences and Technologies, University of Salento, Lecce, Italy; ^3^Department of Agricultural and Biological Engineering, Southwest Florida Research and Education Center, Immokalee, FL, United States; ^4^Department of Physics and Engineering, California State University, Bakersfield, Bakersfield, CA, United States

**Keywords:** convolutional neural networks, deep learning, machine vision, transfer learning, *Olea europaea*, *Xylella fastidiosa*

## Abstract

We have developed a vision-based program to detect symptoms of Olive Quick Decline Syndrome (OQDS) on leaves of *Olea europaea* L. infected by *Xylella fastidiosa*, named X-FIDO (*Xylella* FastIdiosa Detector for *O. europaea* L.). Previous work predicted disease from leaf images with deep learning but required a vast amount of data which was obtained via crowd sourcing such as the PlantVillage project. This approach has limited applicability when samples need to be tested with traditional methods (i.e., PCR) to avoid incorrect training input or for quarantine pests which manipulation is restricted. In this paper, we demonstrate that transfer learning can be leveraged when it is not possible to collect thousands of new leaf images. Transfer learning is the re-application of an already trained deep learner to a new problem. We present a novel algorithm for fusing data at different levels of abstraction to improve performance of the system. The algorithm discovers low-level features from raw data to automatically detect veins and colors that lead to symptomatic leaves. The experiment included images of 100 healthy leaves, 99 *X. fastidiosa*-positive leaves and 100 *X. fastidiosa*-negative leaves with symptoms related to other stress factors (i.e., abiotic factors such as water stress or others diseases). The program detects OQDS with a true positive rate of 98.60 ± 1.47% in testing, showing great potential for image analysis for this disease. Results were obtained with a convolutional neural network trained with the stochastic gradient descent method, and ten trials with a 75/25 split of training and testing data. This work shows potential for massive screening of plants with reduced diagnosis time and cost.

## Introduction

Olive trees are among the most cultivated plants in the world with 10.2 million hectares of planted trees in 2014 (FAO, 2017). In 2013, *Xylella fastidiosa*, a quarantine pathogen known as the causal agent of devastating diseases such as Pierce’s disease or Citrus variegated chlorosis (Chatterjee et al., 2008; Janse and Obradovic, 2010), was discovered in the Apulia region of Italy (Saponari et al., 2013). The pathogen (*X. fastidiosa* subsp. *pauca* strain CoDiRO) was associated to the Olive Quick Decline Syndrome (OQDS or Complesso del Disseccamento Rapido dell’Olivo, CoDiRO) that is causing the collapse and death entire groves of olives in some Apulian districts. Symptoms can vary but, in general, the disease presents itself as leaf scorching, drying, wilting and eventual death. In olive trees, tissue desiccation starts at the tip of the leaves and progresses toward the petiole, soon extending to the whole blade (Martelli, 2016). The first visual symptoms in an infected tree occur between 3 and 18 months after initial infection, depending on the time of year, tree age and variety. In olive trees, the latent period—the period between infection and the appearance of symptoms—will likely provide ample time for the pathogen to spread far away from the initial point of introduction before it is detected, thus large-scale monitoring is desirable. Several diagnostic protocols were tested for the CoDiRO strain (Luvisi et al., 2017), such as ELISA (Loconsole et al., 2014), PCR (Minsavage et al., 1994; Guan et al., 2015), direct tissue blot immunoassay (Djelouah et al., 2014) or loop-mediated isothermal amplification (LAMP) (Harper et al., 2010; Yaseen et al., 2015). Two real-time PCR protocols are also available (Francis et al., 2006; Harper et al., 2010). However, large-scale monitoring such those carried out in Apulia (Martelli, 2016) required a screening of plants to collect samples from symptomatic plants, otherwise the erratic distribution of the pathogen in the host may decrease detection effectiveness of diagnostic tools. Presently, to detect OQDS, a human expert observes the whole canopy (the size of which may be considerable for centenary trees) to detect the symptoms. Because the disease symptoms mainly appear visually on the leaves of an infected plant, computer vision has the potential to provide an effective and fast method for detecting leaf scorch (Luvisi et al., 2016).

**FIGURE 1:**
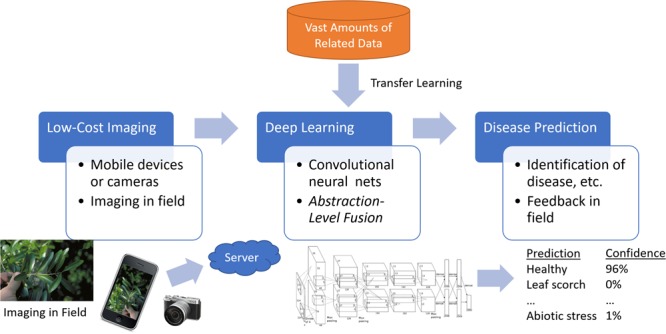
General system overview. A picture of an affected leaf is processed remotely by a server using deep learning. The user receives a report on the diagnosis of the specimen in the field.

We propose a system that would enable growers to take photos of a possibly affected plant with their mobile device, upload the image from the mobile device or computer, have the image processed remotely through the cloud by a deep learning system, and receive a prompt diagnosis of the specimen (**Figure 1**).

In the following, we give a brief overview of the historical basis for quantitative analysis of plants, the development of image-based pattern recognition systems for processing leaves, and the impact of deep learning when applied to pattern recognition systems to identify plants and detect diseases. The earliest work for mathematical analysis of plants dates to a 1936 work by Fisher (1936) that used floral measurements and discriminant analysis to determine the taxonomy of Iris plants. With the advent of LANDSAT technology in the 1970’s, growers were able to quantify the health of whole fields of crops at a glance using satellite imagery (Wiegand et al., 1986). Computer methods for analysis of specific plant specimens appear as early as the 2000’s. Haiyan and He (2005) fuse backpropagation, an artificial neural network designed to imitate the behavior of firing synapses in the human brain, and expert systems, early AI methods based on IF-THEN rules, to pinpoint the origination of nutritional disorders in an orchard. Cunha (2003) applied computer vision techniques to analyze plant leaves imaged with a digital scanner. Computer algorithms automatically calculated the morphological properties of the leaf such as area, perimeter, holes, width and length. Among the earliest works to automatically identify plant species from leaf images dates to a 2001 thesis by Söderkvist (2001). A variety of feature extraction methods (circularity of shape, wavelet transforms, Fourier descriptors, Hu’s moments, etc.) and machine learning methods (support vector machines and backpropagation; Chang and Lin, 2011) were utilized. At best, it was found that backpropagation had mixed success. The data for this work was publicly released and 1,125 leaf images continue to be used as a benchmark dataset.

By the late 2000’s, pattern recognition systems were being proposed that had much better success at automatically identifying the species of plants and presence of disease from leaf images. Pattern recognition is the field of study where a computer automatically predicts the properties of an object from its percepts. The approaches can be categorized into two methodologies: local appearance methods and shape methods. In local appearance methods, the image is processed on a pixel-by-pixel basis. The behavior of a local neighborhood is used to characterize each pixel. Often, a method will quantify the gradient pattern—the strength and direction of the edge at the local pixel. Nilsback and Zisserman (2008) characterized leaf features with the Histogram of Oriented Gradients algorithm (Chen et al., 2014) and the Scale-invariant Feature Transform (SIFT) (Liu et al., 2015). A support vector machine predicted the species of a flower amongst 103 different categories. Xiao et al. (2010) applied Histogram of Oriented Gradients with Maximum Margin Criterion, an algorithm based on discriminant analysis that reduces the number of features to be used by the machine learning algorithm. The algorithm was tested on Soderkvist’s Swedish leaf data set (Söderkvist, 2001) and the Intelligent Computing Laboratory’s (ICL) plant dataset of 17,032 leaf images of 220 different species. In shape methods, also known as segmentation and morphological-based methods, a segmentation algorithm pre-processes the leaf image to obtain an outline of the leaf. From the outline, shape features such as circularity, area, etc. are derived. A machine learning algorithm predicts the disease/species from these features. Munisami et al. (2015) segmented leaf images with Otsu’s algorithm (Baxi and Vala, 2013) and calculated leaf length, width, area, perimeter, hull area, axis length and centroid and applied a K-NN for prediction. The system was tested on 640 images of 32 different plant species.

These methods follow the traditional paradigm of selecting a feature representation based on knowledge of the problem domain. Generally, an image-based pattern recognition system (a) transforms an image to a feature representation that best characterizes the relevant features of an object and (b) a machine learning algorithm carries out the recognition task based on the feature representation. Selecting relevant features in step (a) is a difficult task and features are often selected by empirical analysis. The resulting system is ad-hoc. It will not scale to the detection of other diseases, species or even accommodate great changes in imaging conditions. Often the sensitivity is too low for a production system.

Recently, deep learning has challenged the paradigm of focusing on feature representation. Deep learning may have been used to describe neural networks as early as 1986 (Dechter, 1986) and backpropagation is not a new concept in the field of pattern recognition (LeCun et al., 1989). However, advances in computational power and dissemination of the outstanding performance in the ImageNet challenge (Krizhevsky et al., 2012) have renewed the scientific community’s interest in deep learning (LeCun et al., 2015). A deep learning algorithm learns the features from raw data entirely from the ground up. It combines both steps of a pattern recognition system, (a) and (b) in the previous paragraph. Deep learners have as much as a 99.24% recognition rate for the detection of some plant diseases and species (Mohanty et al., 2016) and show great potential for the field of agricultural engineering. Loosely speaking deep learning is a collection of methods that: (1) improve optimization and generalization of neural networks, and (2) stack layers of transformation to enable the learning algorithm to develop higher levels of abstraction.

A deep learner’s performance increases as the amount of data increases. This enables the system to overcome a variety of imaging conditions, such as lighting conditions, poor alignment, and improper cropping of the object. In contrast, increasing the amount of data for other pattern recognition algorithms (e.g., K-NN, support vector machines) has diminishing returns, and eventual stagnation. However, deep learning is not the end all solution to pattern recognition, as it would appear. The unbounded improvements in accuracy with increasing amounts of data is also deep learning’s greatest disadvantage. A staggeringly large amount of data is required to train a deep learner to exceed a baseline machine learning algorithm’s performance. Unfortunately, in the absence of such large amounts of training data it is inadvisable to train a deep learner from scratch.

Some methods have attempted to address this by borrowing the training data from a related problem domain, called Transfer Learning (Bengio, 2012; Yosinski et al., 2014; Sadeghi and Testolin, 2017). The algorithm is trained on the related problem’s data and then re-trained on data for the problem at hand. The deep learner will learn features relevant to the new problem. If the task is similar enough, such as prediction of plant diseases from images of leaves, the same structures learned by the network will continue to be relevant. This was verified in the field of facial emotion recognition (Kim et al., 2015); deep learners are trained on face identification databases where there is a large amount of data (millions to tens of millions of samples), then later applied to the prediction of facial expression where the data is insufficient to train a deep learner on its own (hundreds to thousands of samples). In this work we will apply this concept to the detection of leaf scorch in *Olea europaea* L.

We also propose a novel deep fusion method that fuses the data with additional features at different levels of abstraction to improve performance when re-applying an already trained deep learner to a new problem. The network will discover relevant low-level features from the raw data to automatically detect veins, colors, and describe events that lead to leaf scorch. Existing applications of deep learning investigated spatial relationships in the image. Fusion, if used, occurred temporally, at the beginning of the processing pipeline, or mixed in the learning process. We will improve upon this with a staggered data fusion scheme that injects relevant features at the increasing levels of abstraction, while allowing the network to discover the complimentary factors leading to disease. Investigating the non-linear relationship found by the network will allow us to better understand the links between plant leaf structure and appearance, disease symptoms.

In the following section, we discuss deep learning methods most similar to our work, and describe how our work is significantly different. The method proposed in this work, Abstraction-Level Fusion, is inspired by the work in Karpathy et al. (2014). In that work, a deep learning neural network is extended to process videos with three modes: early fusion, late fusion and slow fusion. In early fusion, frames are combined as input to the neural network. In late fusion, the additional video frames are injected into the first fully connected later. Slow fusion is a balanced version of both approaches. In Abstraction-Level Fusion, there are multiple levels of fully connected layers. Each fully connected layer receives low-level appearance features such as edge information or order statistics. The method in Karpathy et al. (2014) fuses temporal information, not additional appearance features.

Mohanty et al. (2016) investigated the applicability of two neural networks: AlexNet (Krizhevsky et al., 2012), the seminal neural network that brought deep learning to the attention of the pattern recognition community; and GoogLeNet (Szegedy et al., 2015), an improvement to AlexNet developed by Google that added inception layers to reduce computational burden when carrying out a convolutional neural network. Various transfer learning and image preprocessing methods were tested on a database of 54,306 images of 14 species and 26 diseases obtained from the PlantVillage dataset ^1^. Pawara et al. (2017) carried out a similar study on the Leafsnap (Kumar et al., 2012) and Folio datasets (Munisami et al., 2015), that contained 7,719 and 640 leaf images, respectively. Fujita et al. (2016) applied deep learning to 7,520 cucumber images of 7 different diseases and a healthy control. Unlike other approaches, the approach employed a VGG convolutional neural network (Simonyan and Zisserman, 2015). VGG networks differ from the other two neural network architectures in that the early, filtering layers use much smaller kernels than other networks. We build upon previous work (Fujita et al., 2016; Mohanty et al., 2016; Pawara et al., 2017) by considering improvements to neural network architectures, and we are the first to apply it to leaf scorch detection in *O. europaea* L.

The focus of this paper is a system to process an olive leaf image with computer vision algorithms able to detect OQDS symptoms. We present a novel deep learning framework to organize the learning process into different levels of abstraction. The algorithm discovers low-level features from raw data to automatically detect veins and colors that lead to symptomatic leaves. The system has been implemented as a MATLAB standalone executable for Linux and Mac environments. The proposed system works in the absence of large amounts of training data, a useful feature when samples need to be tested with traditional methods (i.e., PCR) to avoid incorrect input or for quarantine pests which manipulation is restricted.

## Materials and Methods

**FIGURE 2:**
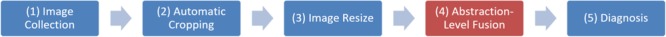
System overview. Red: focus of work.

The system overview is described as follows: (1) for this pilot study, a leaf clipping from a plant to be tested is scanned. In the future, data will be collected of infected leaves in the field. (2) A mask of the leaf is automatically obtained by segmenting the leaf image with Otsu’s algorithm, filtering with a small-window median filter to remove noise, and cropped to the minimally sized bounding box enclosing the segmentation mask. (3) The image is resized to 256 pixels × 256 pixels. All images must be the same resolution to be processed by the machine learning algorithm in the following step. Resizing an image can make it difficult for humans to detect OQDS because it affects symptom presentation (i.e., necrosis and spots). However, so long as the images are resized in a uniform way across all images, it will not inhibit the machine learning algorithm’s ability to learn symptoms. This procedure was performed in related work (Mohanty et al., 2016). (4) The image is processed with the proposed Abstraction-Level Fusion for (5) diagnosis. An overview is given in **Figure 2**.

### Plant Materials

Trials were carried out in orchards located in Province of Lecce (Apulia, Italy), in which OQDS symptomatic and symptomless trees of *O. europaea* L. were monitored. 24 plants grown in *X. fastidiosa* infected areas (in orchards where all plants showed OQDS since 2014, 1 year after first pathogen detection) and 24 plants grown in orchards where the pathogen is not yet detected and non-symptomatic plants were observed. Plants within groups were selected from the same olive tree age (25–30 years), and the same agronomic practices in the last 5 years and similar pedoclimatic conditions.

To evaluate the presence of *X. fastidiosa* in olive trees, sampling was accomplished in relation to symptom expression in October 2016. For each sample of 40 or more leaves, petioles and basal portions of leaf blade were cut with a sterile scalpel. Plant tissue (about 1 g, obtained from 20–25 leaves) was transferred in extraction bags (BIOREBA, Switzerland) for homogenization and 4 mL of extraction buffer was added to each bag (0.2 M Tris – HCl pH 9, 0.4 M LiCl and 25 mM EDTA). Remaining leaves were stored for image acquisition. Homogenization was performed using a semi-automatic homogenizer (Homex 6, BIOREBA) at 50% maximum speed. DNA extraction was performed following Edwards et al. (1991).

TaqMan quantitative PCR protocol with XF-F/R primers and XF-P probe (Harper et al., 2010) was used. Each reaction was prepared using 5 μL from a 20 ng/μL dilution of DNA extracted from 1 g of olive petioles, 200 nM probe, 400 nM forward and reverse primers, in a total volume of 25 μL. The cycling conditions were: 10 min at 95°C, followed by 40 cycles of 95°C for 15 s and 60°C for 1 min with the final dissociation at 95°C for 15 s, 60°C for 30 s and 95°C for 15 s.

*Xylella fastidiosa*-negative samples were also assayed by qPCR for *Verticillium dahliae* (Bilodeau et al., 2012), *Colletotrichum* spp., *Colletotrichum acutatum, C. gloeosporioides* (Garrido et al., 2009) and visually evaluated for presence of *Stictis panizzei, Mycocentrospora cladosporioides*, and *Spilocaea oleagina* or showing disorders caused by abiotic stress. Once health status was confirmed by qPCR and visual assessment, 99 unprocessed leaves from *X. fastidiosa*-positive samples, 100 unprocessed healthy leaves (asymptomatic samples negative to *X. fastidiosa* and other tested/observed pathogen) and 100 unprocessed leaves from *X. fastidiosa*-negative samples but showing other diseases or disorders were scanned for image acquisition (300 dpi).

### Abstraction-Level Fusion

**FIGURE 3:**
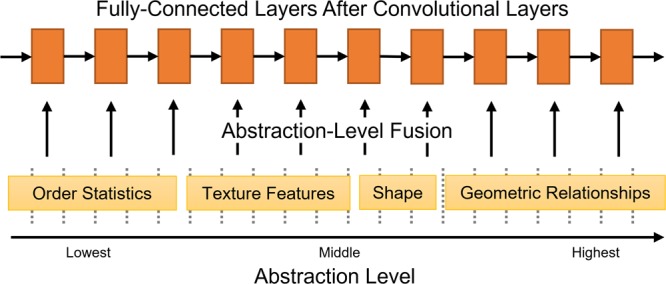
A general overview of the idea of Abstraction-Level Framing. After the convolutional, filtering portion of a convolutional neural network, each fully connected layer receives additional features at increasing levels of abstraction.

A convolutional neural network (LeCun and Bengio, 1995; Cireşan et al., 2011) takes the low-level, pixel representation of an image as input. Each layer is interconnected with the next and is responsible for organizing the visual stimulus into increasingly non-local signals. As the signal passes through successive layers of the network it becomes more complex, and the abstraction-level of the signal increases. This concept inspires us to assist the neural network by injecting features of increasing abstraction-level in the fully connected portion of the network. A general overview of the method is given in **Figure 3**. The idea was originally proposed in a research plan in Rinaldi and Cruz (2016). This work is significantly different; in previous work we used auto-encoders and in this work we use convolutional neural networks. This method is similar to learning with privileged information (Vapnik and Vashist, 2009; Vapnik, 2015), but the method proposed in this paper is based on framing the injection of additional features at different abstraction levels.

We guide the learning process by providing the fully connected layers of the network with high-level image features organized in increasing levels of abstraction; for example, order statistics of the intensity values, edge gradient patterns, image moments from segmentation, etc. With a conventional fully connected layer, we anticipate that the neural network would learn these structures on its own. The key idea with Abstraction-Level Fusion is that, by fusing this information with the network, we hope that the neural network will learn a complimentary organization of signals with the information that is being provided. The full structure is described in **Table 1**. Note that **Figure 3** describes the general idea, and the embodiment of Abstraction-Level Fusion used in this work focuses on texture and shape features.

**Table 1:** Structure of the convolutional neural network employed in this work.

Layer	Type	Abstraction-Level Feature
0	2-D convolutional layer with 11 × 11 kernel, padding size 6, stride length size 4	–
1	Rectified linear activation function layer	–
2	Max pooling layer, downsample factor 2, stride length 2	–
3	2-D convolutional layer with 7 × 7 kernel, padding size 3, stride length size 4	–
4	Rectified linear activation function layer	–
5	Max pooling layer, downsample factor 2, stride length 2	–
6	Fully connected layer, 256 neurons	Edge magnitudes: Grayscaled, original image filtered by Laplacian of a Gaussian, result downsampled by a factor of 8
7	Rectified linear activation function layer	–
8	Fully connected layer, 256 neurons	Shape features: area, perimeter, Hu’s moments (Hu, 1962), Zernike moments (Zhenjiang, 2000)
9	Rectified linear activation function layer	–
10	Fully connected layer, 3 neurons	–
10	Softmax classification layer	–

*Type: the type of layer. Abstraction-Level Feature: The additional features injected in the layer, if any. Features are injected by concatenation with the input from the previous layer.*

### Implementation Details and Parameters

The network is implemented in MATLAB 2016 and carried out on a Dell Precision Rack 7910 with Dual 8-core Intel Xeon E5-2630 v3 processors, 128 GB of DDR4 RDIMM ECC RAM, and a NVIDIA Quadro K6000 12 GB. The network is initially trained on the PlantVillage dataset (Mohanty et al., 2016), then frozen and retrained on the new dataset. Note that the work in Mohanty et al. (2016) uses a pre-trained AlexNet and GoogLeNet neural networks, whereas we train a modified LeNet from scratch on the PlantVillage dataset. Stochastic gradient method is used with a batch size of 60. The base learning rate is 0.01. Step-based learning rate was not employed. Momentum is set to 0.9. Weight decay is set to 0.0005. Gamma is 0.1. For validation, we perform 10 trials splitting the data into random sets of 75% for training and 25% for testing.

We provide a comparison to three baseline methods using various features and a Radial Basis Function (RBF) Support Vector Machine (SVM). For the SVM, γ is set to the inverse of the length of the feature vector and cost c set to 1. The feature vectors are *z*-scored based on the training data before the machine learning step. We compare three feature vector representations: an improved Gabor filter Cruz et al. (2015), Uniform Local Binary Patterns (Almaev and Valstar, 2013), and SIFT features Liu et al. (2015). These features were selected because they provide a good representation of features used currently in literature. For Background Suppressing Gabor Energy Filtering we follow the parameters in Cruz et al. (2015). The whole, filtered image is taken to be the feature vector. For Uniform Local Binary Patterns we use 8 neighbors and a radius of 1. For SIFT Features we follow Liu et al. (2015). SIFT features are extracted sparsely with a stride of 12 pixels in the horizontal and vertical directions. Images are registered by translating the centroid of the segmentation mask to the center of the image. Missing values are filled with repetition. Results are presented with the same validation method (ten random folds with a 75/25 training/testing split).

## Results and Discussion

**FIGURE 4:**
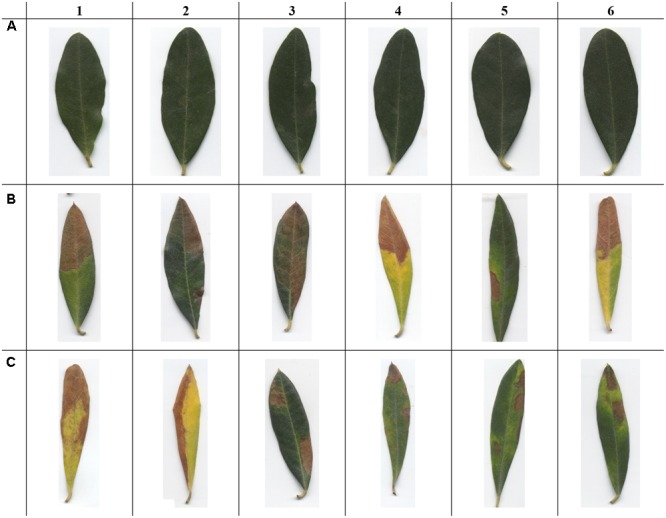
Examples images of *Olea europaea* L. used in this study. **(A)** Healthy control (asymptomatic leaves); **(B)** OQDS-symptomatic leaves (*Xylella fastidiosa*-positive samples); **(C)** *X. fastidiosa*-negative samples showing another pathogen/disorder. Note that the images given here are the native, original aspect ratio of *O. europaea* L. leaves. It is easy to distinguish healthy leaves from non-healthy leaves (**A** vs. **B** or **C**). However, tissue desiccation on the leaf tip, if it occurs, is not exclusive to OQDS (C1, C4, and C6), thus it is challenging to detect the difference between OQDS and non-OQDS samples (**B** vs. **C**).

**FIGURE 5:**
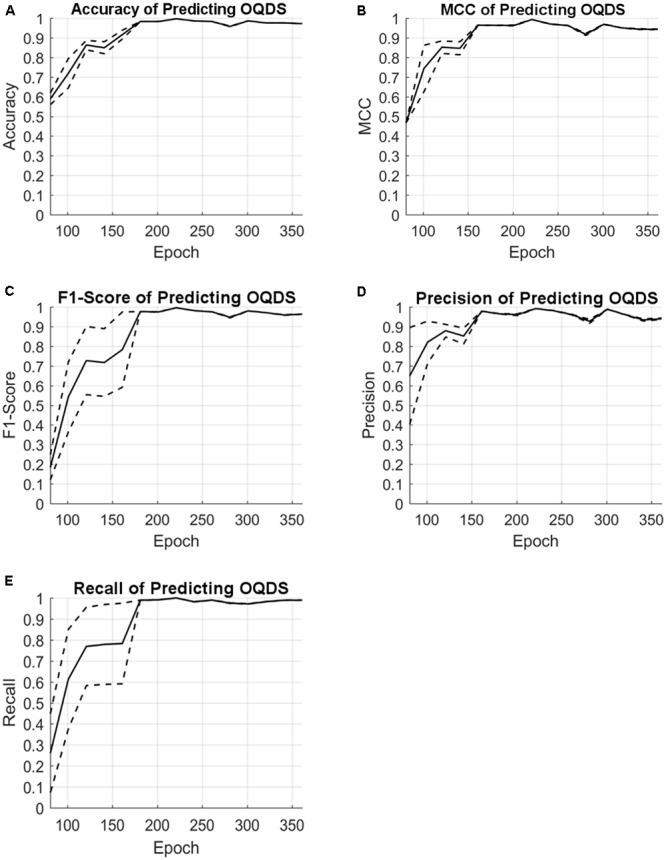
**(A)** Accuracy, **(B)** Matthew’s Correlation Coefficient (MCC), **(C)** F1-Score, **(D)** precision and **(E)** recall of predicting symptoms of Olive Quick Decline Syndrome (OQDS) in images of *X. fastidiosa*-positive leaves of *O. europaea* L. amongst healthy controls (asymptomatic leaves) or *X. fastidiosa*-negative leaves showing other disorders. Higher is better for all metrics.

**FIGURE 6:**
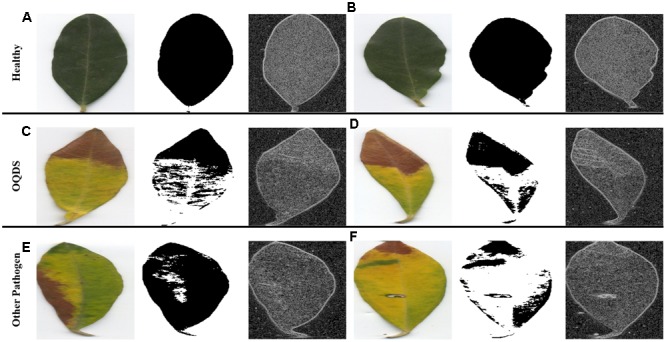
The advantage of including shape and texture. From left to right: The original image, the segmentation map (from which shape features such as moments are extracted), and the edge map. **(A,B)** The healthy leaves (asymptomatic), **(C,D)** OQDS leaves, and **(E,F)** non-OQDS leaves. Healthy leaves do not have any notable features in either the segmentation or edge maps. OQDS results in a more yellow leaf causing a distinct shape in the segmentation map, and note the subtle lines in the dead area of the leaf in **(D)**. While other pathogens/disorders cause yellow leaves, it does not occur as orderly as leaf scorch, and dead areas do not have the distinctive subtle lines as in **(D)**. Note that these images have been resized to 256 × 256 as a part of Step (3) in **Figure 2**.

Example images of healthy leaves, OQDS-symptomatic leaves and leaves showing other disorders are reported in **Figure 4**. In order to stress the proposed detection systems, *X. fastidiosa*-negative leaves showing other disorders (**Figure 4C**) are chosen among samples which may be more easily confused by layperson as OQDS. Results with the proposed system for varying epoch limits are given in **Figure 5**. Numerical results with the proposed system are given in **Table 2A** and baseline results are given in **Table 2B**. The system has exceptional performance despite being trained on only 224 images. Hundreds of data samples are woefully insufficient for training a deep learner, and pretraining the network on the PlantVillage dataset improved performance. Additionally, including edge patterns and shape information contributed to the network’s performance and the advantage can be visualized (**Figure 6**). While it is possible that the network would learn parameters to obtain the segmentation and edge maps in **Figure 6**, providing this information potentially improves convergence because the network does not have to learn these representations from scratch.

**Table 2:** Accuracy, Matthew’s Correlation Coefficient (MCC), F1-Score, Precision and Recall of predicting symptoms of Olive Quick Decline Syndrome (OQDS) in images of *Xylella fastidiosa*-positive leaves of *Olea europaea* L. amongst healthy controls (asymptomatic leaves) or *X. fastidiosa*-negative leaves showing other disorders.

Epoch	Accuracy (%)	Matthew’s Correlation Coefficient (MCC) [-1,1]	F1-Score (%)	Precision (%)	Recall (%)
**(A) Proposed method**			
100	71.93 ± 27.32	0.5419 ± 0.4225	74.37 ± 34.41	82.14 ± 32.62	61.27 ± 48.73
150	84.91 ± 17.44	0.7173 ± 0.4150	84.65 ± 18.13	85.19 ± 20.07	77.87 ± 43.61
200	98.25 ± 2.15	0.9743 ± 0.3320	96.25 ± 4.63	96.05 ± 6.51	99.09 ± 2.03
250	98.60 ± 1.47	0.9811 ± 0.1930	97.02 ± 3.10	98.09 ± 2.62	98.18 ± 2.49
300	98.60 ± 1.47	0.9798 ± 0.2410	96.89 ± 3.45	98.82 ± 2.63	97.18 ± 2.71

**Method**	**Accuracy (%)**	**Matthew’s Correlation Coefficient (MCC) [-1,1]**	**F1-Score (%)**	**Precision (%)**	**Recall (%)**

**(B) Comparison to other methods**			
Background Suppressing Gabor Energy Filtering (Cruz et al., 2015) with RBF-SVM (Chang and Lin, 2011)	63.11 ± 11.91	0.2271 ± 0.2517	65.52 ± 15.15	72.44 ± 14.30	65.28 ± 21.74
Uniform Local Binary Patterns (Almaev and Valstar, 2013) and RBF-SVM (Chang and Lin, 2011)	88.55 ± 16.71	0.7839 ± 0.2936	90.95 ± 11.97	92.12 ± 17.68	92.24 ± 6.16
SIFT Features (Liu et al., 2015) and RBF-SVM (Chang and Lin, 2011)	84.91 ± 17.44	0.7173 ± 0.4150	84.65 ± 18.13	85.19 ± 20.07	77.87 ± 43.61

*Higher is better for all metrics.*

**FIGURE 7:**
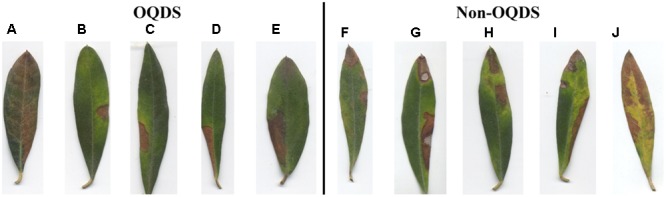
Examples of images that were misclassified more than once across the 10 validation folds. **(A–E)** Images of OQDS leaves. **(F–J)** Images of leaves with pathogens/diseases other than OQDS.

In **Figure 7**, we give examples of images that were misclassified more than once across the ten validation folds. In **Figure 7A**, the desiccated tissue area has a very gentle intensity gradient compared to other images and we suspect that the edge transition from desiccation to healthy tissue is not sharp enough to be detected by the convolutional steps of the neural network. In **Figures 7B–E**, tissue desiccation did not start at the tip of the leaf as expected. However, in the non-OQDS images in **Figures 7F–J**, tissue desiccation appeared at the tip of the leaf resembling OQDS.

### Description of Stand-Alone Executable Program

**FIGURE 8:**
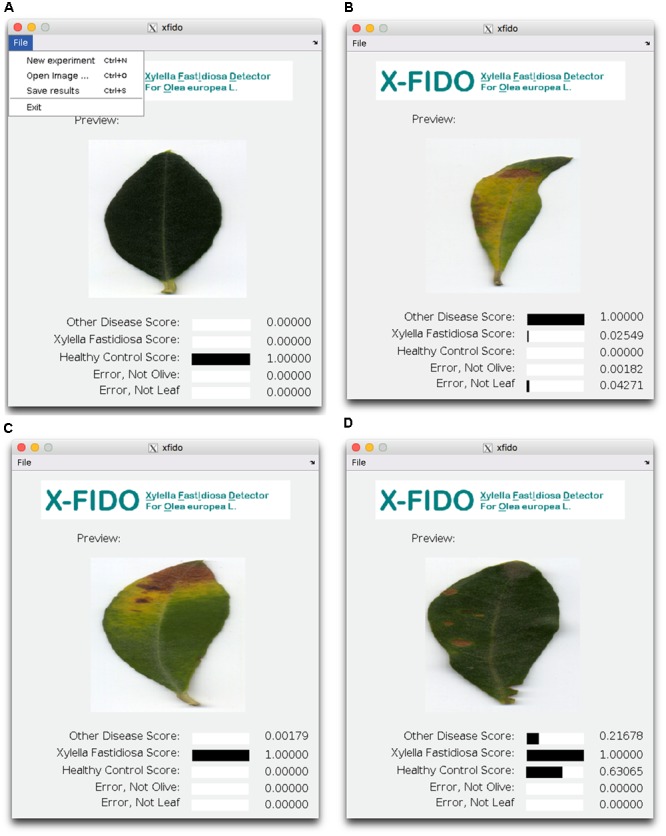
Screen shots of the X-FIDO program. **(A)** The program is simple to operate and consists of three commands: New experiment; Open image, which prompts the user to open an image, automatically processes the image and logs the confidence scores; and Save results, which saves all logged confidence scores to a comma-separated value (CSV) file. In this sub-figure, the program correctly classified a healthy control. **(B)** A non-OQDS pathogen/disorder. **(C)** OQDS (*X. Fastidiosa*). **(D)** A very challenging sample of a non-OQDS image that resembles OQDS because of very faint leaf tip desiccation, and a healthy control because of verdancy.

The model trained in the above experiments is compiled into a stand-alone MATLAB executable file ^2^. The program was developed with the intent to be as simple and intuitive as possible (**Figure 8**). The work flow is described as follows. A user starts a new experiment, and loads images of olive tree to be processed. As each image is loaded, prediction is carried out automatically. A preview of the image is displayed to the screen and confidence scores for each prediction category are displayed to the user. The system detects symptoms of OQDS in images of *X. fastidiosa*-positive leaves of olive tree amongst healthy controls (asymptomatic leaves) or *X. fastidiosa*-negative leaves showing other disorders. It is also capable of detecting two types of errors: (1) the user has given a leaf but it is not *Olea europaea* L., and (2) a general error for when the user has given an image that is not a leaf. Error case (1) was trained on 100 random images of non-olive species from the PlantVillage dataset. Error case (2) is useful for reporting to the user that the system is unsure of the content in the image. It was trained on 100 random images on CalTech 101 (Wang et al., 2006). When the user is finished experimenting on a set of images, the user executes the save option. As the user is processing images the program saves the file name, time, date, and confidence scores for each of the five categories. The “Save results” command stores the saved experimental results to a comma-separated value (CSV) file. To start a new experiment and clear all current experimental results, the user selects “New experiment.”

## Conclusion

Deep learning could revolutionize the field of agriculture, enabling swift, effective methods for the identification of crop diseases from images of leaves (Ampatzidis et al., 2017). Transfer learning enabled the application of deep learning to overcome the lack of sufficient training examples. Large leaf databases such as PlantVillage can be used to enable deep learning for other plant species and diseases where it is too challenging to obtain the tens of thousands of images normally required for deep learning. Collecting a large amount of images may be a difficult task due to unskilled samplers. Furthermore, to efficiently train an automated system for symptom recognition, images should be collected from samples previously tested for pathogen using traditional diagnostic methods, avoiding false positive/negative input. This task may be not easily achievable for large amount of samples or for quarantine pests which manipulation is restricted.

We demonstrate that it is possible to automatically detect leaf scorch in olive tree from leaf clipping images and that it can be discriminated from other disorders or pathogens, despite the strong similarity. However, even if the true positive rate obtained with the proposed method was high, specificity and sensibility of traditional diagnostic methods such as ELISA or qPCR is still unmatched. Thus this method may represent a tool for supporting sampling or could be used to pre-screening of samples before diagnostic tests. Presently, monitoring of OQDS in Europe strongly relies on visual inspection and collection of samples from plants that show standard symptoms, thus the use of *X. fastidiosa*-infected leaves with advanced stage of symptoms is coherent with the aim of the tools. However, the evaluation of the proposed method with earlier symptomatic leaves represents a critical issue that need to be investigated.

We also present a novel algorithm for framing a convolutional neural network, called Abstraction-Level Fusion, that injects additional feature vectors into the network. The layers are abstracted with increasing levels of complexity. In the future, efficacy of the Abstraction-Level Fusion will be applied to other convolutional neural networks such as VGG and inception. We found that the PlantVillage dataset alone was sufficient for transfer learning whereas previous work used pre-trained AlexNet and GoogLeNet networks.

The program shows potential for rapid and automatic detection of OQDS with reduced diagnosis time and cost. Because leaf scorch symptoms present themselves on the leaf when affecting other species, the technologies and algorithms pioneered in this work have broad impact to the detection of *X. fastidiosa* for Pierce’s disease in grapevine or other leaf scorch-related diseases.

## Author Contributions

Conceived and designed the experiments: AC, YA, and AL. Data acquisition of plant pathogen and diagnostic tests: AL and LDB. Processing, analysis and interpretation: AC and YA. Analyzed the data: AC and YA. Prepared the manuscript: AC and YA. Edited the manuscript: AL and LDB.

## Conflict of Interest Statement

The authors declare that the research was conducted in the absence of any commercial or financial relationships that could be construed as a potential conflict of interest.

## Footnotes

1 www.plantvillage.org

2 http://cs.csubak.edu/~acruz/xfido.html
